# Infusing High Value Care Education Directly into Patient Care on the Medicine Wards

**DOI:** 10.15694/mep.2019.000136.1

**Published:** 2019-06-19

**Authors:** Jacqueline M. Schulman, Sonali Palchaudhuri, Brandyn D. Lau, Paul O'Rourke

**Affiliations:** 1 Department of Neurology; 2 Department of Gastroenterology; 3 Johns Hopkins University School of Medicine

**Keywords:** High value care, medical residency, graduate medical education, point of care teaching

## Abstract

This article was migrated. The article was marked as recommended.

*Background:* Multiple national initiatives have been implemented to promote cost-conscious care. Yet, there remains a deficiency of formal high value care (HVC) curricula among internal medicine residency programs.We aimedto develop a curriculum that teaches HVC material that can be utilized at the point of care and to assess the curriculum’s impact on the participants’ attitudes, knowledge, and practice patterns pertaining to HVC.

*Methods:* We conducted our study on the inpatient internal medicine service over two-week rotations at Johns Hopkins Bayview Medical Center. Internal medicine residentsparticipated in two collaborative educational sessions that incorporated an introduction of important concepts in HVC, Bayesian thinking, clinical cases, and a review of a hospital bill of one of the patients under the team’s care. Participants were also encouraged to reflect on their practice patterns and incorporate the HVC principles taught into their daily clinical work. We administered pre- and post-curriculum surveys to assess change in reported HVC-related practice behaviors, knowledge, and attitudes.

*Results:* Forty-seven residents participated in the study. We included the twenty participants who completed both a pre- and post-curriculum survey in the data analysis. After participation in the curriculum, there was a significant increase in the use of pre-test probabilities in clinical decision making ( *p*=0.005). There was also a trend toward improvement in HVC knowledge and practice patterns after the rotation.

*Conclusion:* We implemented a curriculum that may have improved high-value practice patterns through point-of-care education on the inpatient medicine wards.

## Introduction

The United States spends 765 billion dollars annually on potentially avoidable costs, almost a third of which is spent on unnecessary tests and treatments ( Smith, 2012). These unnecessary tests and treatments may lead to patient harm and suffering, such as healthcare-associated infection or increased length of hospital stay ( Badgery-Parker *et al.*, 2019). While a number of national initiatives have been implemented to promote cost-conscious care, most residency programs lack a formal curriculum that provides high value care (HVC) education to trainees ( Cooke, 2010; Hackbarth and Boccuti, 2011; Weinberger, 2011; Colla *et al.*, 2015). When HVC education is provided, it is frequently taught outside of the context of direct patient care through educational methods such as didactic sessions ( Patel *et al.*, 2014). Other programs have attempted a top-down approach for promoting HVC, with limited success ( McMillan and Ziegelstein, 2014). More clinically applicable methods, such as hospital bill reviews, have shown promise ( Nikiforova, 2019).

The purpose of this study was to assess the effect of HVC education on the knowledge, attitudes, and practice patterns of internal medicine resident physicians. In order to introduce formal HVC education in the clinical setting, we developed a curriculum that aimed to teach and practice HVC concepts that were directly related to the care being provided by residents on inpatient internal medicine wards. We explicitly aimed to introduce material and concepts that were related to internal medicine residents’ clinical work and that could be utilized at the point of care. Through completion of the curriculum, we aspired to enhance participant knowledge, attitudes, and practice patterns pertaining to HVC.

## Setting and Participants

This study was conducted on the general internal medicine inpatient service at Johns Hopkins Bayview Medical Center, where one of the four inpatient medicine house staff teams was designated the HVC team. The curriculum was implemented in January 2017; our study began at curriculum initiation and lasted 12 months through December 2017. Our study population consisted of internal medicine residents of all training levels who were assigned to the HVC inpatient medicine ward team for a two-week rotation block. In total, out of 54 residents in the program, 47 distinct residents rotated through the HVC team and participated in our curriculum. The study was deemed exempt by the Johns Hopkins Medicine Institutional Review Board.

## Curriculum Description

Prior to the start of each two-week HVC inpatient medicine rotation, new residents and attendings rotating onto the HVC team received curricular objectives. Two collaborative, team-based, hour-long HVC educational sessions were organized on the team’s non-admitting days, with one session per week. The two educational sessions were held in the team workroom at a pre-determined time to avoid interfering with clinical duties. Each session was facilitated by a member of our HVC training team, consisting of volunteer faculty and clinical fellows with interest in HVC. A Microsoft PowerPoint presentation, adapted from the American College of Physicians’ (ACP) HVC Curriculum for Educators and Residents, was developed and used in the sessions ( Clancy C, 2016). The presentations were designed to standardize the key learning objectives but with prompts to encourage group participation and reflection.

**Figure 1.**
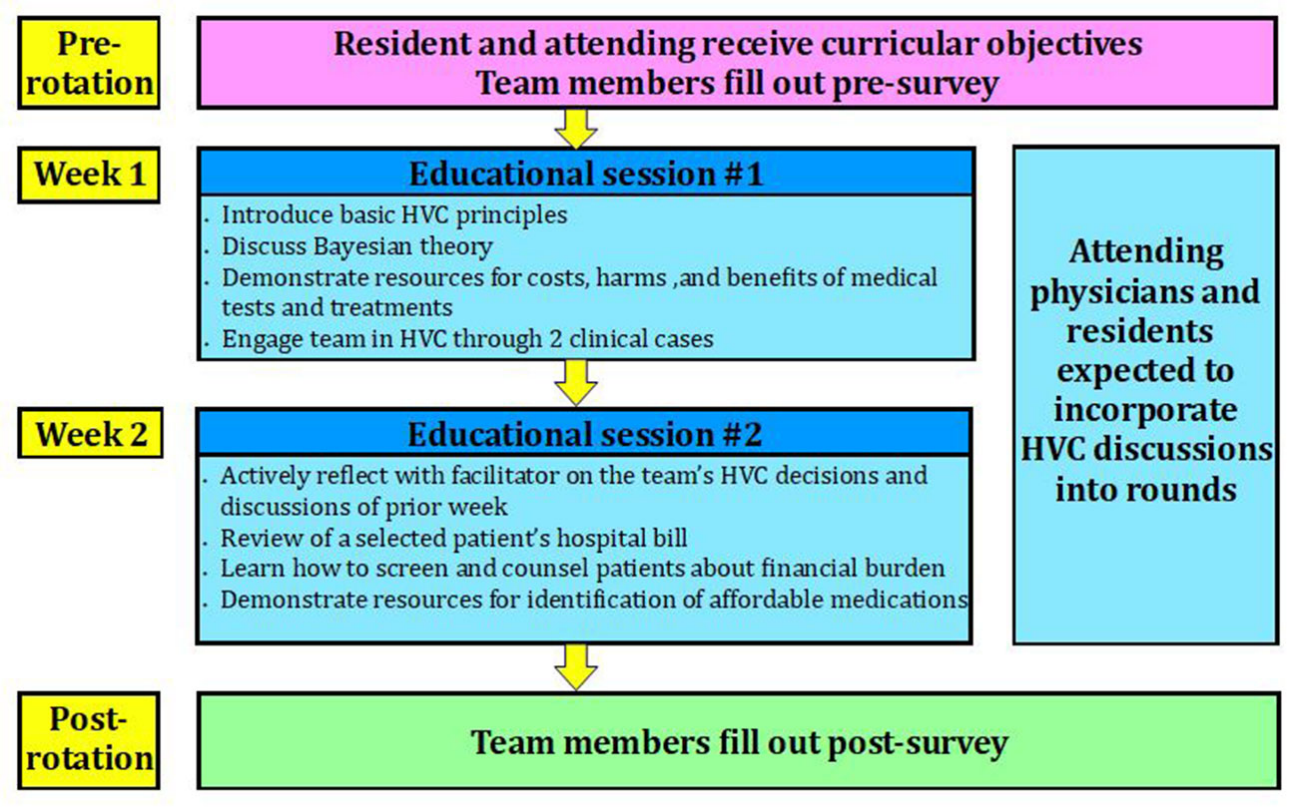
Organization flow chart of a high value care curriculum on an inpatient medicine house staff team

Figure 1 demonstrates the organization of our HVC curriculum on the inpatient medicine house staff service. The sessions utilized HVC educational exercises and resources to teach the principles and importance of HVC and equip participants with the skills to incorporate HVC into their direct patient care. In the first educational session, learners were introduced to principles of HVC, resources that provide information on costs, harms, and benefits of medical tests and therapies, and Bayesian thinking. They also engaged in two clinical cases adapted from the ACP website that demonstrated several key HVC principles ( Clancy C, 2016). During this initial interactive session, the participants were encouraged to incorporate the principles taught, such as the use of pre-test probabilities, into their daily patient rounding discussions and clinical decisions. At the end of the hour, the team was asked to select a patient under their care whose hospital bill they were interested in evaluating.

In the second facilitated educational session, the team was guided through shared reflection of the care they had provided during the rotation and discussed whether and how Bayesian thinking and HVC principles were applied during their daily work. The team then reviewed the itemized hospital bill for the patient they had previously chosen. The bill review allowed the team to discuss any surprising charges, to assess whether and how the tests and treatments ordered impacted the care of the patient, and to propose alternative approaches for future patients as appropriate. In addition, team members learned how to assess and counsel individual patients about financial burden using the GOTMeDS mnemonic ( Kumar R, 2016). Participants were then assigned the task to practice this skill at the bedside and engage at least one of their patients in a conversation related to costs of care. The residents and attending on the HVC team were encouraged to incorporate the HVC principles addressed during the two facilitated educational sessions into daily work rounds and in the direct care of their patients.

## Curriculum Evaluation

We developed and administered an online survey to assess resident knowledge, attitudes, and practice patterns pertaining to HVC before and after their participation in the HVC rotation between January 2017 through December 2017.

Participants were asked to complete anonymous pre- and post-rotation 11-item and 12-item surveys respectively via Qualtrics®. The surveys utilized questions assessing attitudes using a 5-point Likert scale (strongly agree, somewhat agree, neither agree nor disagree, somewhat disagree, strongly disagree), knowledge questions using both multiple choice and free text formats, as well as questions assessing practice patterns with answer options that included a frequency scale from 0 to ≥4. Comparisons between responses before and after the rotation were done using Chi-squared tests.

## Results

Surveys were collected from 37 residents before the rotation and 27 residents after the rotation. The response rate was 79% (37 of 47 unique house staff) in the pre-rotation survey and 57% (27 of 47 house staff) in the post-rotation survey. Of note, there were several residents who filled out the pre-rotation survey but not the post-rotation survey, and vice versa. Thus, to provide an accurate comparison between the two groups, we included only the 20 participants who completed both surveys in the data analysis (43% or 20 of 47 total residents). Out of the 20 participants, 65% were in post-graduate year (PGY) 1, 20% were in PGY 2, and 15% were in PGY 3.

**Figure 2.**
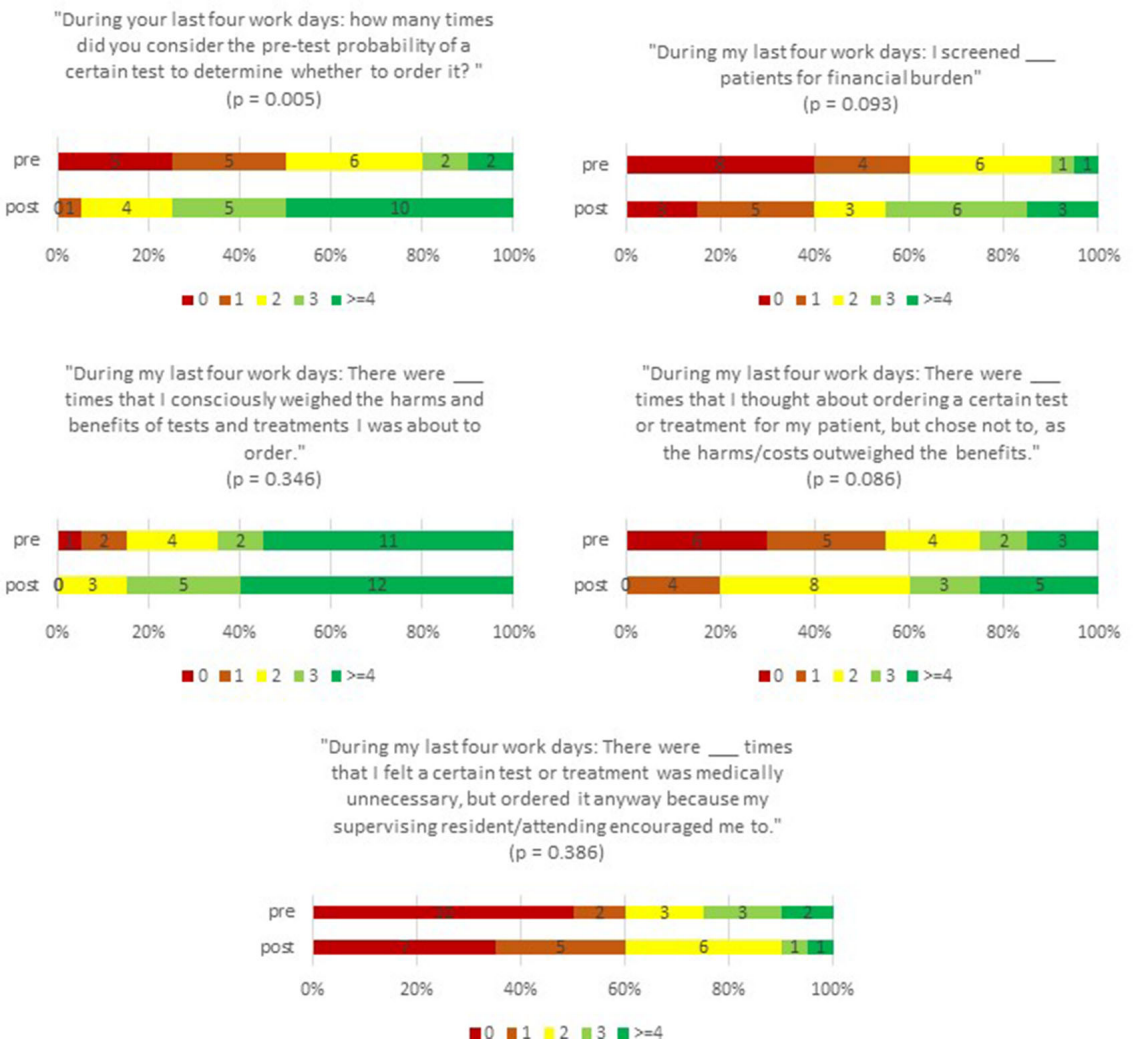
Comparison of resident practices pertaining to high value care pre- and post-implementation of a high value care curriculum

Figure 2 compares resident self-reported practices pre- and post-rotation on the HVC team. Residents reported a statistically significant increase in their use of pre-test probability in their decisions to order tests after the rotation (p=0.005). Although the difference in pre- and post-curriculum values were not statistically significant in the other survey questions, there was a trend towards improved HVC practice patterns after participation in the curriculum.

Survey questions that assessed HVC attitudes did not demonstrate significant differences among participants before and after the curriculum. In the knowledge questions using free text answer format, participants were asked to name resources they would use to explore the approximate cost of tests and treatments. In the pre-rotation survey, 64% of the responses were either left blank or mentioned Google, with the remaining responses naming resources such as GoodRx®, hospital resources, UpToDate®, and Choosing Wisely®. In the post-rotation survey, only 38% of the responses were left blank or mentioned Google, with the remaining responses expanded to include resources such as RxPriceQuotes® and ACR Appropriateness Criteria® in addition to the resources named in the pre-rotation survey. They were also asked to state the three questions they would use to screen their patients for financial burden (“Do your medications cost too much? Have you ever cut back on medications because of cost? Have you ever cut back on other things (e.g. food, leisure) due to high medication costs?”). The percent of participants who could name at least two out of the three questions improved from 10% prior to the curriculum to 55% after the curriculum.

## Discussion

In our study, internal medicine residents who completed our HVC curriculum reported a significant increase in the use of pre-test probabilities in clinical decision-making and demonstrated trends in improvement in other HVC practice patterns. Although a growing number of residency programs are developing HVC curricula, it is unclear how to effectively incorporate HVC teaching at the point of patient care ( Sommers *et al.*, 2012; Post *et al.*, 2013; Stammen *et al.*, 2015; Cayea *et al.*, 2018). Implementing a HVC curriculum into the daily workflow of medical trainees is challenged by time, clinical demands, and competing content that must also be taught during residency. We developed a curriculum that was concise and could be incorporated into an inpatient medicine rotation in order to deliver maximum value for the participants. Facilitated reflection of residents’ clinical decisions and review of a patient’s hospital bill provided an opportunity for feedback on the team’s practice habits. The combination of education with regular audit and feedback of practice habits for medical residents has also been shown to significantly improve behavior both among those who are direct targets of education as well as among clinicians who closely interact with recipients of education and feedback ( Lau *et al.*, 2016a; Lau *et al.*, 2016b).

There were several limitations of our study. First, the small sample size of our study (due in part to the size of the residency program) affected the ability to identify significant changes in our measures. The positive direction of all measures suggests that this HVC curriculum should be tested on a larger population. Second, the study was conducted in a single institution and specialty, and thus the results may be less generalizable. However, the broad measures are likely applicable to other settings. Third, while the surveys assessed resident perceptions of HVC practice patterns, we did not objectively assess practice behaviors. Finally, variation in completion of evaluation surveys may have led to reporting bias. Finally, residents who were rotating later during the study period may have also been aware of the lessons from the curriculum, as they had rotated with team members who had been exposed to the curriculum. This may have led to bias in the pre-curriculum surveys.

## Conclusion

Teaching HVC principles and resources to medical trainees has tremendous potential to influence their care of patients. Our curriculum provided residents with HVC education at the point of patient care and led to an increased frequency of self-reported high value practice behavior. Future studies of our HVC curriculum at different training programs are warranted. In addition, further studies are necessary to evaluate the effect of HVC education on actual test and therapy ordering practices of medicine residents and to assess the longitudinal effect of these curricular interventions.

## Take Home Messages

• Effective high value care (HVC) curricula are lacking in most residency programs
• We developed a HVC curriculum focusing on point of care education
• Comparison of pre- and post-curriculum survey responses showed a significant increase in the use of pre-test probabilities in participants’ clinical decision-making
• There was a positive trend toward improvement in knowledge and practice behaviors pertaining to HVC

## Notes On Contributors

Dr. Jacqueline Schulman is a neurology resident at Massachusetts General Hospital and Brigham and Women’s Hospital (Harvard Medical School). She completed her internal medicine preliminary year at Johns Hopkins Bayview Medical Center. She has a special interest in autoimmune neurological diseases such as multiple sclerosis, as well as in medical education and high value care.

Dr. Sonali Palchaudhuri is a gastroenterology fellow at the University of Pennsylvania. She completed her internal medicine residency at Johns Hopkins Bayview Medical Center. She is interested in quality improvement, high value care, and medical education and plans to pursue a career in general gastroenterology.

Brandyn Lau is Assistant Professor of Radiology and Radiological Science and Health Sciences Informatics at the Johns Hopkins School of Medicine, Director of High Value Imaging Informatics in the Johns Hopkins Health System, and Director of Informatics for the High Value Practice Academic Alliance.

Dr. Paul O’Rourke is Assistant Professor of Medicine and Associate Program Director of the Johns Hopkins Bayview Internal Medicine Residency Program. He is the High Value Care Physician Lead for the Department of Medicine at Johns Hopkins Bayview Medical Center.

## Declarations

The author has declared that there are no conflicts of interest.

## Ethics Statement

This study has been deemed exempt by the Johns Hopkins Medicine Institutional Review Board (Reference Number IRB00122217).

## External Funding

This article has not had any External Funding
