# Supplementary material for: Infusing High Value Care Education Directly into Patient Care on the Medicine Wards
Source: MedEdPublish (2016). 2019 Jun 19;8:136. [Version 1] doi: 10.15694/mep.2019.000136.1 (PMC10712453; doi:10.15694/mep.2019.000136.1)
Supplement: Peer review history

# Reviewer report: Reviewer response for version 1

**DOI:** 10.21956/mep.19844.r29932

**Published:** 2020-02-22

**Authors:** Ken Masters

**Affiliations:** 1 Sultan Qaboos University

This review has been migrated. The reviewer awarded 4 stars out of 5 An interesting article on infusing high-value care (HVC) education directly into patient care on the medicine wards. The paper gives a good description of the need for implementing HVC and shortcomings of current methods of education in this area, and the paper describes in suitable detail the methods in which the HVC was incorporated into the students’ rotation. The success of the intervention is well-illustrated in the students’ behaviour pattern changes (albeit self-reported, rather than directly observed) and knowledge.Within the limitations of the study (chiefly, single-centre with a small sample), the authors have done well to produce a paper that is certainly worth reading. I look forward to a more detailed study on the topic, with a larger sample.

Reviewer Expertise:

NA

No decision status is available

# Reviewer report: Reviewer response for version 1

**DOI:** 10.21956/mep.19844.r29930

**Published:** 2019-07-22

**Authors:** Trevor Gibbs

**Affiliations:** 1 AMEE

This review has been migrated. The reviewer awarded 3 stars out of 5 Thank you for asking me to review this paper. It is an interesting paper to review, particularly by a UK graduate who is used to evidence-based healthcare and having curricula with a large element of clinical decision making and critical thinking built into the undergraduate curriculum. Some would anticipate, and even argue that the NHS is not a cost-conscious service, but even in the NHS measures are taken to avoid over-reliance on tests and investigations. However, and despite these comments, I did enjoy the paper. I wondered if it had a US bias and I think that this could have been nullified by a slightly longer introduction that explored the importance of health economics, the relevance of cost-consciousness and expand on the term of high -value healthcare- a term still I feel in its infancy in many parts of the world, despite the increasing number of papers on the subject.The paper was well written and the research valid. It is never surprising that a pre / post test and a self-perception evaluation gives the answers that are present here, but I feel that the authors recognised this limitation of their research- together with the small number of participants and the possibility of bias introduced through group mixing. I would hope that the authors consider a longer evaluation looking at effects once the participants were in independent practice. This is a good start to an interesting piece of educational work, which hopefully can show enough effect, and to have such influential activities, that it becomes standard practice. I think however that there is still work to be done.

Reviewer Expertise:

NA

No decision status is available

# Reviewer report: Reviewer response for version 1

**DOI:** 10.21956/mep.19844.r29929

**Published:** 2019-07-19

**Authors:** Gregory Seymann

**Affiliations:** 1 University of California San Diego

This review has been migrated. The reviewer awarded 4 stars out of 5 In this small single center study, the authors describe a novel approach to teaching High Value Healthcare by applying the core QI technique of “Just In Time” education to curriculum delivery on the inpatient Medicine service. In contrast to common approaches that typically consist of a detached standalone didactic curriculum, the investigators integrated the curriculum into the weekly activities of the ward team and measured the impact in real time. The small sample size and single center design limited the authors’ ability to demonstrate impact of the curriculum on attitudes and practices, but their unique approach, which included review of a hospital bill for one of the team’s patients to assess the value of the treatments provided, remains an important contribution to the education and high value care literature. Although limited to an Internal Medicine service, the techniques described are generalizable across disciplines.

Reviewer Expertise:

NA

No decision status is available

# Reviewer report: Reviewer response for version 1

**DOI:** 10.21956/mep.19844.r29931

**Published:** 2019-07-06

**Authors:** ANEESH SINGHAL

**Affiliations:** 1 MGH

This review has been migrated. The reviewer awarded 5 stars out of 5 In this excellent paper, Schulman and colleagues report the results of a well designed study showing significantly improved clinical decision making by residents participating in a HVC education program. A major quality-improvement challenge in academic hospitals is to educate the "front line" residents and staff about clinical utilization management especially when it comes to high-cost testing. The results of this study suggest feasibility and application across a broad range of academic hospitals and departments -- embracing this approach would have enormous impact in education as well as improving the cost and quality of care.

Reviewer Expertise:

NA

No decision status is available

# Reviewer report: Reviewer response for version 1

**DOI:** 10.21956/mep.19844.r29933

**Published:** 2019-07-01

**Authors:** Anthony Accurso

**Affiliations:** 1 Family Health Centers of NYU Langone Brooklyn

This review has been migrated. The reviewer awarded 4 stars out of 5 Schulman et al write a helpful and practical piece on how an internal medicine program can approach the task of instructing its trainees on how to provide high-value care. Their approach - to label a specific 2-week wards rotation as the “high-value care-focused” rotation, allows time for thoughtful practice, coaching and reflection. As such, this model seems well positioned to develop the ‘skill’ of high-value practice, rather than simply imparting the knowledge. The increase in frequency of application of pre-test probability consideration and screening for financial burden are evidence that the skills were being developed over the 2-week rotation.

Reviewer Expertise:

NA

No decision status is available
